# *Astragalus membranaceus* polysaccharide (APs) and Eugenol: Multi-target Anti-inflammatory, Antioxidant, Antimicrobial, and anticancer effects validated by in Silico studies

**DOI:** 10.1016/j.jgeb.2025.100646

**Published:** 2026-01-02

**Authors:** Mohamed Khedr, Ahmed E.M. Abdelaziz, Fatima Albadwi, Fady Sayed Youssef, Eman M. Abd El-maksoud, Alsayed E. Mekky, Ebrahim Saied, Mohamed A.M. El-Tabakh, Eslam S Abdelmouty, Jayda G. Eldiasty, Mohammad Y. Alfaifih, Ali A. Shatii, Serag Eldin I. Elbehairii, Mohammed Aufy

**Affiliations:** a Department of Botany and Microbiology, Faculty of Science, Al-Azhar University, 11884 Nasr, Cairo, Egypt; b Botany and Microbiology Department, Faculty of Science, Port Said University, 23 December Street, P.O. Box 42522, Port-Said, Egypt; c State Key Laboratory of Bioreactor Engineering, R&D Center of Separation and Extraction Technology in Fermentation Industry, East China University of Science and Technology, Shanghai 200237, China; d Department of Pharmacology, Faculty of Veterinary Medicine, Cairo University, Giza 12211, Egypt; e Biochemistry Department, Faculty of Veterinary Medicine, Alexandria University, Alexandria, Egypt; f Physiology and Biochemistry Department, Faculty of Veterinary Medicine, Ain Shams University, Egypt; g Zoology Department, Faculty of Science, Al-Azhar University, Cairo, Egypt; h Biology Department, University College of Haqel, University of Tabuk, Saudi Arabia; i Central Labs, King Khalid University, Alqura’a, Abha, Saudi Arabia; j BiologyDepartment, Faculty of Science, King Khalid University, Abha, Saudi Arabia; k Tissue Culture and Cancer Biology ResearchLaboratory, King Khalid University, Abha, Saudi Arabia; l Department of Pharmaceutical Sciences, Division of Pharmacology and Toxicology, University of Vienna, Vienna, Austria

**Keywords:** *Astragalus membranaceus*, Antioxidant, Anti-inflammatory, Multidrug-resistant (MDR), Anticancer activity, Eugenol

## Abstract

*Astragalus membranaceus* is a traditional medicinal plant with diverse therapeutic properties largely attributed to its polysaccharides (APs). This study evaluated the antimicrobial, anti-inflammatory, antioxidant, and anticancer activities of APs and eugenol, both individually and in combination, against multidrug-resistant (MDR) pathogens and HepG2 liver cancer cells. Thirty bacterial and ten *Candida* isolates were recovered from skin abscesses, with five identified as MDR strains (*Staphylococcus haemolyticus, S. aureus, E. coli, Acinetobacter baumannii*, and *Candida auris*), confirmed by 16S rDNA and ITS sequencing. Both APs and eugenol exhibited marked antimicrobial activity, while their combination achieved the strongest inhibition (up to 27.3 ± 0.4 mm). *C. auris* was highly sensitive to APs alone (MIC: 2 ± 0.2 µg/mL). The combination also significantly downregulated IL-6, IL-17, and TNF-α levels, and showed potent COX-2 inhibition (0.10 ± 0.01 µg/mL), surpassing celecoxib (0.9 ± 0.05 µg/mL). Antioxidant analysis (DPPH assay) revealed superior radical scavenging by the combination (57.5 ± 1.3 % %). Molecular docking confirmed the activity of eugenol, showing favorable binding to DNA gyrase B, sterol demethylase, COX-2, xanthine oxidase, and caspase-3, with the strongest affinity for xanthine oxidase (−5.25 kcal/mol). In anticancer assays, eugenol induced dose-dependent inhibition of HepG2 cell proliferation, while APs displayed limited cytotoxicity. Notably, the combination reduced cell viability to 3.77 ± 0.4 % % at 400 µg/mL, consistent with apoptotic changes. Collectively, these findings highlight the synergistic potential of APs and eugenol as a multi-target therapeutic approach against MDR infections, inflammation, oxidative stress, and liver cancer.

## 1 Introduction

Antibiotics have long been the cornerstone of therapy for bacterial infections and remain vital in the management of serious illnesses such as typhoid, pneumonia, and tuberculosis [1]. However, their effectiveness has been undermined by the rapid emergence of resistant strains, largely driven by excessive and inappropriate use. Despite ongoing efforts to develop new antibiotics, resistance often arises shortly after their introduction, resulting in the spread of multidrug-resistant (MDR) pathogens [2]. This alarming trend has prompted global health authorities, including the World Health Organization (WHO), to classify antimicrobial resistance as one of the top three threats to public health. Of particular concern are the ESKAPE pathogens (*Enterococcus, Staphylococcus, Klebsiella, Acinetobacter, Pseudomonas, Enterobacter,* and *Escherichia coli*), which are responsible for a large proportion of hospital- and community-acquired infections and display high levels of resistance [3]. These challenges highlight the urgent need to explore novel antimicrobial agents and alternative therapeutic approaches to overcome the growing burden of MDR infections [4]. Infection, along with injury or exposure to harmful agents, is a key trigger of inflammation, which represents a fundamental protective mechanism of the body against such risks [5]. This defense process involves immune system activation, production of pro-inflammatory mediators, and subsequent tissue repair. However, dysregulated or chronic inflammation contributes to the development and progression of several pathological conditions, including autoimmune diseases, metabolic disorders, and cancer. A clear understanding of the molecular mechanisms underlying inflammatory responses is therefore critical for designing effective therapeutic strategies 6, 7. Classically, inflammation is characterized by five hallmarks: heat, redness, swelling, pain, and loss of function. Increased blood flow accounts for redness and heat, while fluid accumulation leads to swelling 8, 9. On the molecular level, toll-like receptors (TLRs) are type I transmembrane proteins that detect pathogen-associated molecular patterns (PAMPs), such as bacterial and viral DNA, lipoproteins, flagellin, lipopolysaccharides, and single-stranded RNA [10]. TLRs are expressed by various cell types, including fibroblasts, epithelial cells, and immune cells, and initiate signaling pathways that recruit neutrophils and macrophages, stimulate cytokine and antimicrobial peptide production, and ultimately activate adaptive immunity [11]. Epigenetic regulation has also been implicated in inflammation, as demethylation of the TLR2 promoter is linked to early upregulation of angiogenic markers and pro-inflammatory cytokines [12]. Cytokines further shape the inflammatory environment. Pro-inflammatory mediators include IL-1β, IL-6, IL-12, IL-18, interferon-gamma (IFN-γ), and tumor necrosis factor (TNF), while anti-inflammatory cytokines such as IL-4, IL-10, IL-13, and IL-19 are secreted to counterbalance excessive responses. Elevated levels of IL-1β, IL-6, and TNF in older adults have been correlated with disease burden, frailty, and mortality, with IL-6 in particular often described as “the cytokine for gerontologists” playing central roles in metabolic regulation, the acute-phase response, and chronic disease pathogenesis [13]. Furthermore, IL-17 family members are recognized as potent pro-inflammatory cytokines involved in autoimmune conditions such as ankylosing spondylitis and psoriasis [14].

New strategies should be concerned with challenges like MDR and Cancer. One of these is *Astragalus membranaceus* is one of the most popular medicinal legumes which are used worldwide. It is mainly originates in China and is known as “Huangqi.” and is used as medicine and food additive to revitalize the spleen [15]. The *Astragalus membranaceus* root contains many biologically active constituents such as polysaccharides and saponins which have been widely used in traditional medicine for many years as a promoter of immunity [16]. Astragalus polysaccharide (APS), which is derived from stems or dried roots of *A. membranaceus* is a water-soluble heteropolysaccharide that possesses biological functions [17]. It is considered the most vital natural active compound in *A. membranaceus* that possesses many pharmacological functions [16]. In consequence of its low side effects and biohazards, in addition to non-residual and non-tolerance functions [18]. Recent studies have shown that *Astragalus membranaceus* root attain antioxidant, anti-inflammatory, along with antiviral and antimicrobial functions with growth-promoting and immunity-enhancement activities as mentioned by some authors 19, 20. Improving immunity and enhancing the synthesis and production of cytokines and immunoglobulin by the active product; APS, as well as having a cytotoxic effect on tumor cells by boosting tumor cell apoptosis and down-regulating cancer cell propagation [21]. Besides APs, Eugenol comes in our second choice, which is an essential oil that is well-known for its antimicrobial, anti-inflammatory, and antioxidant effects [22]. It is widely used in a variety of industries, such as food, medicines, and cosmetics, and is well known for its antibacterial qualities. The molecular formula C_10_H_12_O_2_, which is particularly 4-allyl-2-methoxyphenol, describes its chemical structure. Its hydrophobic properties and capacity to rupture bacterial membranes, resulting in antibacterial action, are facilitated by this structure [23].

Cancer remains a major global health burden, with liver cancer ranking among the most aggressive and fatal malignancies. Although advances in chemotherapy, targeted agents, and immunotherapies have improved treatment options, their clinical use is often hampered by severe toxicity, limited tolerance, and the rapid emergence of resistance [24]. These drawbacks have prompted growing interest in natural bioactive compounds, which are generally associated with lower toxicity and diverse pharmacological properties. Astragalus polysaccharides (APs), isolated from *A. membranaceus*, are well-documented for their immunomodulatory, antioxidant, and anti-inflammatory functions, and recent studies suggest promising antitumor potential [25]. Likewise, eugenol, a phenolic constituent of clove oil, has attracted attention due to its broad antimicrobial, antioxidant, and anti-inflammatory activities, alongside evidence of cytotoxic effects against several cancer cell lines [26]. In this context, the present study was designed to assess the antimicrobial, antioxidant, and anti-inflammatory properties of APs and eugenol, both individually and in combination, while also evaluating their cytotoxic activity against HepG2 liver cancer cells. In addition, molecular docking analyses were conducted to elucidate potential mechanisms underlying their antimicrobial and anti-inflammatory actions.

## 2 Materials and methods

### 2.1 Materials

Eugenol (≥99 % purity, catalog no. E51791) was sourced from Sigma-Aldrich (St. Louis, MO, USA). Astragalus polysaccharide (APS, 100 %) was obtained as a commercial preparation (Mega Immune® Poultry Immune Booster; Shijiazhuang ZDHF Stock-raising Co., Ltd., Hebei, China). α-Chymotrypsin (Type II, lyophilized powder, 40 U/mg protein; catalog no. C4129) derived from bovine pancreas was purchased from Sigma-Aldrich (St. Louis, MO, USA).

### 2.2 Isolation, purification, and identification of pathogenic microbes

Between August 2024 and May 2025, clinical isolates were collected from dermal abscesses of fifty hospitalized patients (20 males and 30 females) at Benha University Hospitals. This research was conducted in accordance with the ethical standards outlined in the Declaration of Helsinki and received approval from the Institutional Review Board of Alexandria University (IRB approval no. AU13020920240133; 2 September 2024). Prior to specimen collection, all participants provided written informed consent, and their personal data were handled and stored with confidentiality. Specimens were immediately transported to the laboratory of the Medical Microbiology and Immunology Department, where they were cultured on CLED agar for bacterial isolation and potato dextrose agar [27] for *Candida* recovery. Following 24 h of incubation, isolates were purified and identified on the basis of Vitek system reports and antibiotic susceptibility testing. Multidrug-resistant (MDR) strains were subsequently selected for further analysis [28].

### 2.3 Molecular identification of MDR bacterial and fungal isolates

The predominant MDR isolates selected for 16S rDNA analysis included *Staphylococcus haemolyticus, Staphylococcus aureus, Escherichia coli,* and *Acinetobacter baumannii*. Genomic DNA was extracted using the Wizard Genomic DNA kit (Promega, Madison, WI, USA), and the 16S rDNA region (∼1500 bp) was amplified with primers RW and 16Sb FW under standard PCR conditions [29]. Amplicons were confirmed by agarose gel electrophoresis, purified (QIAquick kit, Qiagen, USA), and sequenced using a Perkin Elmer 377 DNA sequencer [4].

For fungal identification, genomic DNA from the main *Candida* isolate (Candida auris, identified by VITEK2) was amplified under optimized PCR cycling conditions. The PCR products were visualized on agarose gels, purified, and further analyzed using universal ITS primers (ITS-1 and ITS-4) [30].

### 2.4 Antimicrobial activity

The antimicrobial activity of APS, eugenol, and their 1:1 (v/v) mixture was tested by agar well diffusion against C*. auris, S. aureus, S. haemolyticus, E. coli*, and *A. baumannii*. The isolates, previously identified using microbiological and molecular methods, were grown overnight to logarithmic phase, and cell densities were adjusted to 0.5 McFarland (∼10^8^ CFU/mL for bacteria and ∼ 10⁶ CFU/mL for fungi). Standardized inocula were spread on Mueller–Hinton agar (bacteria) or Sabouraud Dextrose agar (*C. auris*). Wells (6 mm) were filled with 100 μL of APS, eugenol, or their combination at selected concentrations. Gentamicin (10 μg/disc) and fluconazole (25 μg/disc) served as positive controls, while broth and broth with DMSO were used as negative and solvent controls, respectively. Plates were incubated at 37 °C for 24 h (bacteria) or 30 °C for 48 h (C. auris). Antimicrobial efficacy was expressed as inhibition zone diameters (mm), including the well diameter 31, 32.

#### 2.4.1 Determination of MIC and MLC for bacterial and fungal strains

The MIC and MLC of eugenol, APS, and their 1:1 (v/v) mixture were assessed by an INT-based broth microdilution assay, with separate protocols for bacteria and fungi. Bacterial inocula (5 × 10^5^ CFU/mL in CAMHB) were exposed to serial two-fold dilutions of the test agents in 96-well plates and incubated at 37 °C for 24 h. After adding INT (0.2 mg/mL), wells without color change indicated growth inhibition [29]. The MIC was recorded as the lowest concentration achieving ≥ 90 % inhibition, confirmed at OD_595_, while the MLC was determined by subculturing aliquots from growth-free wells onto TSA and checking for colony formation after 24 h [33].

For *C. auris* (PP967949), suspensions were adjusted to 1–2 × 10^3^ CFU/mL in RPMI-1640 medium (MOPS-buffered, pH 7.0) and inoculated into plates containing serial dilutions of the test compounds. Cultures were incubated at 30 °C for 24–48 h, followed by INT addition [34]. The MIC was defined as the lowest concentration with complete growth suppression. MLC values were confirmed by subculturing onto SDA plates and incubating for 48 h. Gentamicin (bacteria) and fluconazole (*C. auris*) were used as positive controls, while broth and broth with DMSO served as negative and solvent controls, respectively [35].

### 2.5 Modulation of cytokine gene expression in RAW 264.7 Macrophages

Total RNA was isolated from RAW 264.7 macrophages after 24 h of treatment under different experimental groups: negative control (LPS-stimulated, 0.5 mg/mL), APS (2–8 mg/mL), eugenol (3–11 mg/mL), APS–eugenol mixture (v:v, based on the most effective doses), and a positive control with chymotrypsin (0.2–1 mg/mL). RNA extraction was performed with TRIzol reagent (Gibco), followed by cDNA synthesis using the Advantage RT-for-PCR Kit (Clontech). RT-PCR reactions (25 μL) contained 75 ng cDNA, 200 μM dNTPs, 0.1 μM primers, 1.5 mM MgCl_2_, and 1 U Taq polymerase (Takara). Specific primers for IL-10 and IL-17 were used, with β-actin serving as the internal control (primer sequences in Table 1). The cycling protocol consisted of 94 °C for 4 min; 32 cycles of 94 °C for 30 s, 50 °C for 50 s, and 71.5 °C for 1 min; followed by a final extension at 71.5 °C for 5 min. PCR products were resolved on agarose gels (TAE buffer, ethidium bromide staining), visualized under UV illumination, and compared against a 100 bp DNA ladder (MBI Fermentas) [36].

**Table 1:** Specific primers which are used in two anti-inflammatory markers through RT-PCR reaction.

Primer	Sequence 5–3	Length	Tm	GC%	Strand	Ref.
IL-17 FW	GGGAAGTTGGACCACCACAT	20	54	55	+	[37]
IL-17 RW	TCTCCACCCGGAAAGTGAA	19	53	51	−	[37]
IL-10-FW	CTCCGAGATGCCTTCAGCAG	20	54	55	+	This study
IL-10-RW	AGAAATCGAAGACAGCGCC	19	53	54	−	
b-actin FW	CATTGCTGACAGGATGCAGAAGG	23	54	55	+	
b- actin RW	TGCTGGAAGGTGGACAGTGAGG	22	54	55	−	
IL-6-FW	TACCACTTCACAAGTCGGAGGC	22	55	55	+	
IL-6-RW	CTGCAAGTGCATCATCGTTGTTC	23	54	55	−	
TLR2-FW	GCTCAGACTTGAGCACTATACA	22	57.5	46	+	[38]
TLR2-FW	GGCTTGAACCAGGAAGACGA	20	59.9	55	−	

#### 2.5.1 Reaction conditions for Real-Time PCR

Semiquantitative PCR was carried out using cDNA templates and the primers listed in Table 1. Each 25 µL reaction mixture contained 50 ng cDNA, 25 pmol of each primer, 12.5 µL of 2 × QuantiTect SYBR® Green RT Mix (Fermentas), and RNase-free water. After brief centrifugation, reactions were run on a Rotor-Gene 6000 system (Qiagen, USA). The amplification program for the target genes consisted of an initial denaturation at 95 °C for 2 min, followed by cycles of 95 °C for 30 s, 57.5 °C for 30 s, and 72 °C for 30 s. For the reference gene 18S, optimized cycling conditions were 95 °C for 2 min, 44 °C for 25 s, and 72 °C for 30 s. Data interpretation was based on threshold cycle (CT) values [38].

### 2.6 Biochemical Assessment of antioxidant capacity via DPPH radical scavenging

The antioxidant potential of APs, eugenol, and their combination (APs–eugenol) was assessed through the DPPH (2,2-diphenyl-1-picrylhydrazyl) free radical scavenging assay. Different concentrations of each sample (0–1000 μg/mL) were prepared, and 3 mL of the test solution was mixed with 1 mL of 0.1 mM DPPH in ethanol. The mixtures were kept in the dark at room temperature for 30 min, after which absorbance was recorded at 517 nm using a UV–Vis spectrophotometer (Milton Roy). Ascorbic acid served as the reference standard [39]. Radical scavenging activity (%) was calculated according to the following equation:

Effect of DPPH scavenging (%) or percent inhibition (%) = A0 − A1/A0 × 100.

where: A1 was the absorbance when the test or reference sample was present, and A0 was the control reaction's absorbance [39].

### 2.7 Enzymatic inhibition of cyclooxygenase isoforms (COX-1 and COX-2)

The COX inhibitory activity of APS, eugenol, and their combination (APS–eugenol, 1:1 v/v) was assessed using a commercial colorimetric COX inhibitor screening kit (Cayman, Kit No. 560131; Cayman Chemical, USA). Celecoxib was used as the positive control [40]. In brief, the reaction mixture contained 150 µL of assay buffer, 10 µL of heme, 10 µL of either COX-1 (ovine) or COX-2 (human recombinant) enzyme, and 20 µL of each test sample at its IC_50_ concentration. For the combined treatment, the IC_50_ values of APS and eugenol were mixed in equal proportions. After gentle shaking (20 s), the plate was incubated for 5 min at 25 °C. The reaction was then initiated by adding 20 µL of the colorimetric substrate solution along with arachidonic acid, followed by 10 min incubation at 25 °C. Absorbance was recorded at 590 nm using a microplate reader [41].

### 2.8 Protein preparation and molecular docking study

Eugenol structure was built in the mol file format using the chem draw program. PDB structures of the five target proteins [DNA Gyrase B (5L3J), Sterol demethylase (5FSA), Cyclooxygenase-2 (COX-2)(5KIR), xanthine oxidase (3B9J), and caspase-3 (2XYP)] are downloaded from the protein data bank (http://www.rcsb.org.pdb) 42, 43. Molecular docking simulations were performed to evaluate binding affinities and interaction patterns, with validation conducted through re-docking of co-crystallized ligands to ensure computational reliability 44, 45.

### 2.9 Mitochondrial-Dependent cytotoxicity in HepG2 cells (MTT Assay(

The cytotoxic effects of APS, eugenol, and their combination (APS–eugenol) on human hepatocellular carcinoma cells (HepG2, ATCC HB-8065) were assessed using the MTT assay [46]. This assay measures cell viability based on the conversion of MTT to purple formazan by mitochondrial dehydrogenases in living cells. HepG2 cells were cultured in 96-well plates and incubated at 37 °C for 24 h before treatment. Cells were then exposed to varying concentrations of APS, eugenol, or APS–eugenol (25–400 µg/mL). After treatment, the medium was removed, and 100 µL of MTT solution (0.5 mg/mL in culture medium) was added, followed by 2 h incubation at 37 °C. Formazan crystals were solubilized using 100 µL of 10 % SDS, and absorbance was measured at 560 nm with a microplate reader 47, 48.

## 3 Statistical analysis

All experiments were performed in triplicate, and data were expressed as mean ± SD. Statistical analysis was conducted using one-way ANOVA with Tukey’s post-hoc test, considering p < 0.05 as significant.

## 4 Results and discussion

### 4.1 Isolation and identification of pathogenic microbes

A total of 30 bacterial and 10 Candida isolates were recovered from skin abscess samples collected from patients at Benha University Hospital. All isolates were purified and identified using Gram staining and the VITEK 2 automated system. Among the bacterial isolates, four strains *K. pneumoniae*, *A. baumannii*, *S. haemolyticus*, and *S. aureus* exhibited MDR profiles. Additionally, one *Candida* isolate showed resistance to multiple antifungal agents as illustrated in Table 2. These findings are consistent with several recent studies reporting high rates of MDR organisms in skin and wound infections, especially in hospital environments. For instance, Alharbi, [49], reported similar patterns of resistance in *K. pneumoniae* and *S. aureus* isolated from wound swabs in tertiary care hospitals. The rising prevalence of MDR *Acinetobacter* and *Staphylococcus* species has been attributed to the overuse of broad-spectrum antibiotics and inadequate infection control protocols [50]. Furthermore, the detection of a multidrug-resistant *Candida* isolate may indicate the emergence of non-albicans species such as Candida auris, which is known for its resistance to azoles and amphotericin B [51]. This aligns with findings by Ahmad and Alfouzan, [52], who documented the spread of fluconazole-resistant *C. auris* in nosocomial infections with skin involvement. To confirm the identities of these resistant strains, molecular identification was carried out using 16S rDNA sequencing for bacterial isolates and ITS region amplification for the *Candida* isolate. This molecular confirmation ensures accurate species-level identification and supports previous recommendations for integrating molecular diagnostics in routine hospital surveillance [53].

**Table 2:** Identification and characterization of selected multidrug-resistant clinical isolates recovered from skin abscess samples.

Isolate Code	Species	Gram Stain	Resistance Profile	VITEK ID
B01	*Klebsiella pneumoniae*	−ve	MDR	Confirmed
B02	*Acinetobacter baumannii*	−ve	MDR	Confirmed
B03	*Staphylococcus haemolyticus*	+ve	MDR	Confirmed
B04	*Staphylococcus aureus*	+ve	MDR	Confirmed
F01	*Candida* spp.	Yeast	Multidrug resistant	Confirmed

Note: Isolate codes were assigned as B for bacterial strains and F for fungal strains.

### 4.2 Molecular identification of MDR microbes

**Fig. 1:**
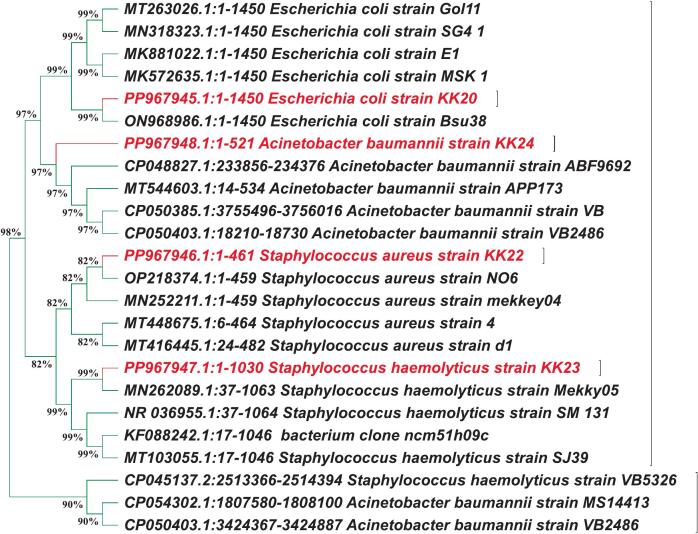
Phylogenetic tree of four bacterial mdr isolates were tested in this study based on their 16s rdna gene sequencing analysis through ncbi blast.

**Fig. 2:**
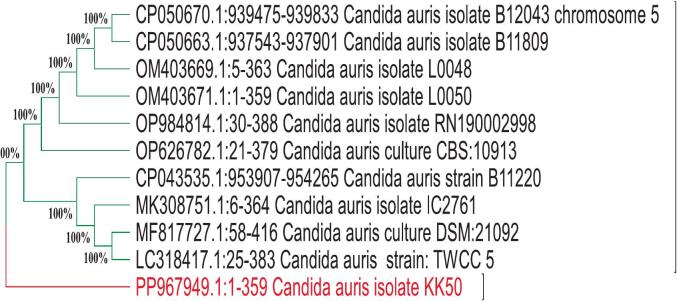
Phylogenetic tree of *Candida auris* [PP967949] tested in this study based on its 18S rDNA gene sequencing analysis through NCBI BLAST.

The molecular identification of the most extensive MDR bacterial isolates via 16S rDNA sequencing confirmed the presence of *Staphylococcus haemolyticus* [PP967947], *Staphylococcus aureus* [PP967946], *Escherichia coli* [PP967945], and *Acinetobacter baumannii* [PP967948], as shown in Fig. 1. These bacterial species are well-documented for their clinical significance and increasing resistance to multiple antibiotic classes, particularly in hospital-acquired infections. Previous studies have reported the emergence of MDR *A. baumannii* and *S. aureus* as major threats in nosocomial settings due to their capacity to form biofilms and acquire resistance genes 54, 55. Hassan et al. [56] identified the isolates based on molecular analysis using 16S rRNA and physiological characteristics through the VITEK 2 automated system. The bacterial strains were deposited in GenBank with the following accession numbers: *S. haemolyticus* MST1 (KY550377), *P. aeruginosa* MST2 (KY550378), *K. pneumoniae* MST3 (KY550379), *E. coli* MST4 (KY550380), and *E. coli* MST5 (KY550381). Such MDR isolates may reflect excessive or inappropriate antibiotic use in clinical settings, leading to selection pressure that favors resistant strains [57]. In parallel, the only extensively resistant fungal isolate was identified by ITS sequencing as *Candida auris* [PP967949] (Fig. 2), a multidrug-resistant yeast species that has recently emerged as a global health concern. The identification was further validated through BLASTn alignment on NCBI. *C. auris* is known for its persistence in hospital environments and its resistance to common antifungal agents such as fluconazole and amphotericin B [52]. The phylogenetic analyses provided further support for the species-level identification and emphasized the genetic similarity of our isolates to previously reported strains from clinical origins[58].

### 4.3 Antimicrobial activity

**Fig. 3:**
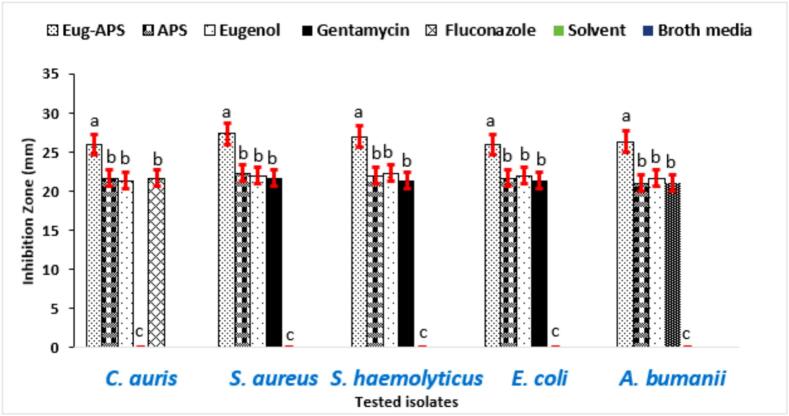
Antimicrobial activity of APS, eugenol, and their combination against MDR bacteria and *C. auris*. Gentamicin and fluconazole served as positive controls, while broth and DMSO were used as negative and solvent controls. Data represent mean ± SE of three independent experiments. Different letters above the bars indicate statistically significant differences (p < 0.05, one-way ANOVA followed by Tukey’s post hoc test).

By applying the two natural products, APS and eugenol, individually beside their combination (v:v), noticeable antimicrobial activity was observed against all tested MDR microorganisms. Notably, the combination treatment exhibited the most potent inhibitory effect compared to either compound alone, suggesting a potential synergistic interaction between APS and eugenol (Fig. 3). The most significant inhibition zones were recorded as follows: *Staphylococcus aureus* (27.3 ± 0.4 mm), *Staphylococcus haemolyticus* (27.0 ± 0.3 mm), *Acinetobacter baumannii* (26.3 ± 0.5 mm), Escherichia coli (26.0 ± 0.4 mm), and *Candida auris* (26.0 ± 0.3 mm). When used individually, APS demonstrated higher activity against *C. auris* (21.7 ± 0.3 mm) and *S. aureus* (22.3 ± 0.2 mm), while eugenol was more effective against *S. haemolyticus* (22.3 ± 0.3 mm), *E. coli* (22.0 ± 0.2 mm), and *A. baumannii* (21.7 ± 0.4 mm). Negative (broth only) and solvent (broth with DMSO) controls showed no inhibitory activity against the tested pathogenic organisms. All experiments were performed in triplicate (n = 3), and values are expressed as mean ± SE. Statistical analysis revealed that the combination treatment exhibited significantly higher inhibitory activity compared to either APS or eugenol alone across all tested MDR strains (p < 0.05).

These findings highlight the broad-spectrum antimicrobial potential of both natural compounds and point to their differential target specificity, possibly due to differences in membrane structure and resistance mechanisms among the tested species [59].

The enhanced efficacy observed with the combination suggests that APS and eugenol may act through complementary mechanisms, enhancing membrane permeability or interfering with multiple metabolic pathways simultaneously [60]. Similar synergistic effects have been reported in previous studies, where combining natural bioactive compounds led to increased efficacy and lower resistance development 61, 62. Supporting this, a recent study by Di Consiglio et al. [63] demonstrated that the presence of phenol groups in polymer side chains significantly contributed to radical scavenging and antimicrobial activities against *S. epidermidis*. This aligns with our findings, where the phenolic compound eugenol contributed to substantial antimicrobial activity, likely through similar redox-related and structural disruption mechanisms. For example, poly(ethylene-co-vinyl acetate) (EVA) films incorporated with citronellol, eugenol, and linalool have been developed and tested for their antimicrobial and antibiofilm activities against *L. monocytogenes, S. aureus, S. epidermidis, E. coli*, and *P. aeruginosa* under both mono- and dual-species conditions [64]. The potent antimicrobial effect of eugenol observed in this study is consistent with evidence reported in earlier investigations. For instance, Abdou et al. [65] reported that eugenol and its two synthetic derivatives acetyleugenol and epoxyeugenol demonstrated potent inhibitory effects against *E. coli* and *S. aureus*, reinforcing the efficacy of phenolic structures in antimicrobial strategies. These results underscore the potential application of such combinations as alternative or adjunct antimicrobial therapies, especially in light of the growing threat posed by MDR pathogens including *C. auris*, which is often recalcitrant to conventional antifungal agents.

#### 4.3.1 MIC of APS, eugenol, and their combination

**Fig. 4:**
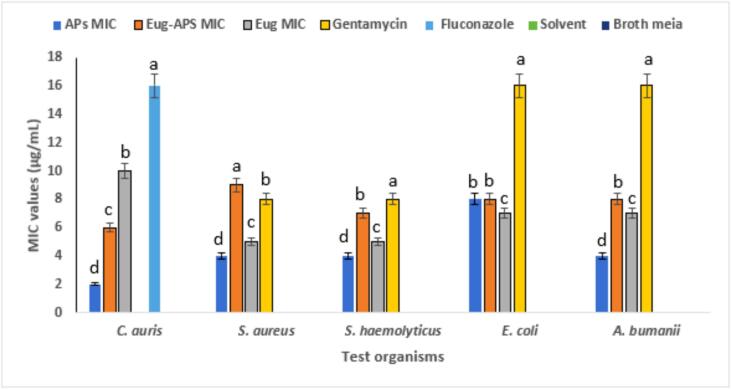
MICs of APS, eugenol, and their combination against selected pathogens. Gentamicin and fluconazole served as positive controls, while broth and DMSO were used as negative and solvent controls. Data represent mean ± SE of three independent experiments. Different letters above the bars indicate statistically significant differences (p < 0.05, one-way ANOVA followed by Tukey’s post hoc test).

The MIC values presented in Fig. 4 demonstrate the antimicrobial efficacy of the three tested compounds APs, eugenol, and their combination against a range of pathogenic bacteria and fungi. These values were compared with standard antibiotics such as gentamicin and fluconazole, which served as positive controls. A negative control (broth only) and a solvent control showed no inhibitory activity against the tested pathogenic organisms. *C. auris*, a multidrug-resistant fungal pathogen, showed notable susceptibility to APs with an MIC of 2 ± 0.2 µg/mL, which is significantly lower than that of fluconazole (16 ± 0.5 µg/mL). This suggests the potential of APs as a promising alternative in antifungal therapy. Similar findings were reported by Hetta et al. [66], who found that *C. auris* typically exhibits fluconazole MICs ranging from 16 to > 64 µg/mL, confirming resistance patterns and emphasizing the need for effective alternative agents.

For *S. aureus*, the APs–eugenol combination (MIC = 9 ± 0.3 µg/mL) and eugenol alone (MIC = 5 ± 0.2 µg/mL) showed strong antibacterial effects, whereas APs alone displayed a moderate effect (MIC = 4 ± 0.2 µg/mL), which was comparable to gentamicin (MIC = 8 ± 0.4 µg/mL). These findings are consistent with those of Pacyga et al. [67], who reported MIC values between 4 and 10 µg/mL for various plant-derived compounds against Gram-positive bacteria. Similarly, for *S. haemolyticus*, APs and eugenol achieved MICs of 4 ± 0.3 and 5 ± 0.2 µg/mL, respectively, again approaching the activity of gentamicin. Comparable results were also described by Wang et al. [68], where *Ginkgo biloba* exocarp extract displayed potent inhibitory effects against clinical isolates, with MICs of 2 µg/mL for *S. epidermidis*, 4 µg/mL for *S. haemolyticus*, and 8 µg/mL for *E. faecium*. Collectively, these observations emphasize the promise of phytochemicals as alternatives or complements to conventional antibiotics in the treatment of resistant Gram-positive infections.

*E. coli*, a Gram-negative bacterium known for its robust outer membrane, showed slightly higher MIC values across all formulations (7–8 ± 0.3 µg/mL), with gentamicin showing higher activity (MIC of 16 ± 0.5 µg/mL). Nevertheless, the tested plant-based compounds still achieved effective inhibition. Attaallah Ibrahim and Kadhim Mohammed, [31] reported that 99.3 % of uropathogenic *E. coli* isolates exhibited multidrug resistance. The MIC of eugenol ranged from 1.25 to 5  μg/mL, while that of Fosfomycin was 512 to 1024  μg/mL. The MBC values were 5–10  μg/mL for eugenol and 2048  μg/mL for Fosfomycin. The combination showed a clear synergistic effect, as 1/4 MIC of eugenol reduced the MIC of Fosfomycin to 1/8. Most isolates were inhibited within 4–8 h by eugenol, 8–12 h by Fosfomycin, and 4–8 h by the combined treatment. For *A. baumannii*, a highly resistant nosocomial pathogen, moderate susceptibility was observed with MIC values ranging between 4 ± 0.2 and 8 ± 0.3 µg/mL. All experiments were performed in triplicate (n = 3), and values are expressed as mean ± SE. Statistical analysis using one-way ANOVA followed by Tukey’s post hoc test revealed significant differences among the tested compounds, with the combination treatment exhibiting the highest activity across all MDR strains (p < 0.05). Overall, the tested plant-derived compounds, particularly in combination, exhibited potent antimicrobial activity against both Gram-positive and Gram-negative bacteria as well as fungal pathogens. Their efficacy, especially against multidrug-resistant strains, underscores the therapeutic promise of natural products. The observed effects may be attributed to known mechanisms of action of plant phytochemicals, including disruption of microbial cell walls, interference with membrane permeability, and inhibition of metabolic enzymes.

#### 4.3.2 MLC of APs, eugenol, and their combination

**Fig. 5:**
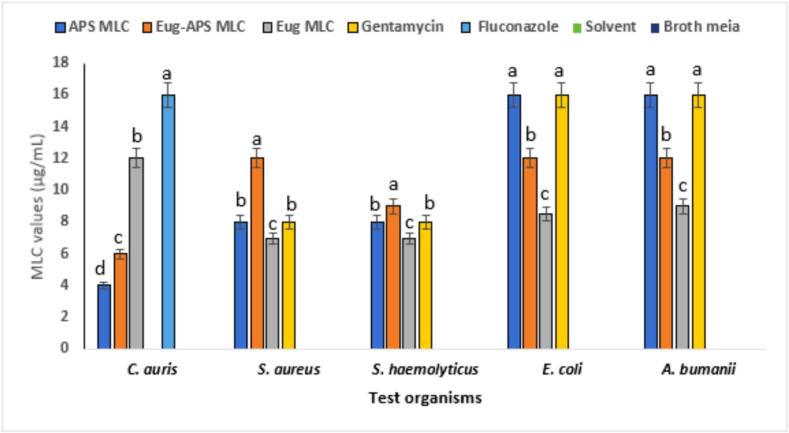
MLC values of APS, eugenol, and their combination against pathogens. Gentamicin and fluconazole served as positive controls, while broth and DMSO were used as negative and solvent controls. Data represent mean ± SE of three independent experiments. Different letters above the bars indicate statistically significant differences (p < 0.05, one-way ANOVA followed by Tukey’s post hoc test).

The MLC values presented in Fig. 5 demonstrate the bactericidal/fungicidal potential of APS, APS-eugenol (Eug-APS), and eugenol (Eug) against a panel of Gram-positive, Gram-negative, and fungal pathogens. Among all tested agents, eugenol exhibited the highest MLC value against *C. auris* (12 ± 0.3 µg/mL), indicating moderate fungicidal activity. In contrast, APS and Eug-APs showed relatively better activity, with MLCs of 4 ± 0.2 and 6 ± 0.3 µg/mL, respectively, against *C. auris*, suggesting synergistic or enhanced effects on the fungal cell wall. In bacterial strains, the MLC of APS against *S. aureus* and *S. haemolyticus* was 8 ± 0.2 µg/mL, which is equivalent to gentamicin (8 ± 0.3 µg/mL), while the MLC values of Eug-APs and eugenol were higher (up to 12 ± 0.3 µg/mL), indicating slightly reduced efficacy. Against *E. coli* and *A. baumannii*, APS and Eug-APs had higher MLCs (16 ± 0.4 and 12 ± 0.3 µg/mL, respectively), suggesting comparatively lower activity against Gram-negative bacteria, possibly due to the outer membrane acting as a barrier. Negative (broth only) and solvent (broth with DMSO) controls showed no inhibitory activity against the tested pathogens. All experiments were performed in triplicate (n = 3), and values are expressed as mean ± SE. Statistical analysis using one-way ANOVA followed by Tukey’s post hoc test revealed significant differences among the tested compounds, with the combination treatment generally exhibiting enhanced bactericidal/fungicidal activity (p < 0.05). The results align with previous findings. For instance, de Sousa Eduardo et al., [69] reported that eugenol exhibited MLCs ranging from 8–16 µg/mL against *S. aureus* and *E. coli*, supporting the values observed in our study. A study by He et al., [70] demonstrated that Astragalus polysaccharides showed strong killing activity against *S. aureus* with an MLC of 10 µg/mL, which is comparable to our findings. Furthermore, Li et al., [71] indicated that APs can compromise bacterial membrane integrity, leading to cell lysis, particularly in *S. aureus* and *P. aeruginosa* strains, consistent with the MLC values obtained here.

### 4.4 Modulation of Pro-Inflammatory cytokine gene expression

**Fig. 6:**
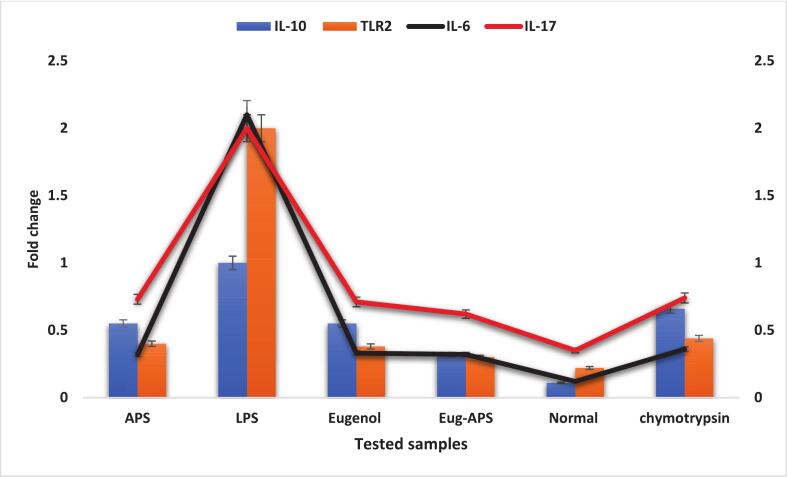
Fold-change of gene expression of IL-6, IL-17, and TNF-α in APS, eugenol, and APS–eugenol treated groups, normalized to the housekeeping beta-actin gene. Data represent mean ± SE of three independent experiments. Statistical significance among treatments was assessed using one-way ANOVA followed by Tukey’s post hoc test (p < 0.05).

The effects of APS and eugenol on pro-inflammatory cytokine gene expression were evaluated through transcriptional profiling of IL-6, IL-17, and TNF-α (Fig. 6). All experiments were performed in triplicate (n = 3), and data are presented as mean ± SE. Statistical significance was determined using one-way ANOVA followed by Tukey’s post hoc test (p < 0.05, p < 0.01, p < 0.001). All treated groups exhibited significant suppression in mRNA expression relative to the positive control (chymotrypsin). The most pronounced effect was observed with the APS–eugenol combination, which reduced IL-6, IL-17, and TNF-α expression to 0.23 ± 0.02-fold (c), 0.59 ± 0.03-fold (b), and 0.36 ± 0.02-fold (b), respectively, confirming potent synergistic inhibition of inflammatory signaling. When tested individually, APS markedly suppressed IL-6 (0.32 ± 0.02-fold, b) and IL-17 (0.73 ± 0.03-fold, a), whereas TNF-α reduction was moderate (0.98 ± 0.04-fold, a). Eugenol alone produced a similar transcriptional suppression, decreasing IL-6 and IL-17 to 0.33 ± 0.02-fold (b) and 0.71 ± 0.03-fold (a), while TNF-α expression remained only slightly affected (0.92 ± 0.03-fold, a). Negative controls showed no change in cytokine expression.

Different letters above the values indicate statistically significant differences among treatments (p < 0.05). These results indicate that APS and eugenol independently downregulate pro-inflammatory gene activity, and their co-administration potentiates the effect through cooperative molecular interactions.

These outcomes indicate that APs and eugenol independently downregulate pro-inflammatory gene activity, and their co-administration potentiates the effect by cooperative molecular interactions. Previous biochemical reports support these findings. Plant-derived nanostructures and phenolic compounds were shown to modulate cytokine expression and attenuate NF-κB signaling, in agreement with the observed sensitivity of IL-6 and IL-17 to both agents. The limited suppression of TNF-α further reflects the relative resistance of this pathway to phenolic modulation. Collectively, the data demonstrate that the APs–eugenol system acts on transcriptional regulators of inflammation, providing a molecular rationale for their anti-inflammatory synergy.

These results are in agreement with multiple prior studies. Dai et al. [72] reported that silver nanoparticles synthesized using *Andrographis paniculata* extracts were effective in downregulating IL-6 and IL-17 in LPS-induced inflammation models, supporting the current results of APs alone. Additionally, Gojani et al. [73] documented the anti-inflammatory role of eugenol, showing that it can reduce IL-6 and IL-17 expression in activated macrophages, which aligns with the reductions observed in our study. Interestingly, TNF-α levels were more resistant to change in both our study and previous literature, indicating that IL-6 and IL-17 are more sensitive markers to eugenol-based treatments [74]. Furthermore, Puja et al. [75] highlighted the enhanced anti-inflammatory potential achieved by combining plant-based nanoparticles with phytochemicals. Their study showed that such combinations could suppress a broader spectrum of inflammatory mediators more effectively than individual treatments. This synergistic behavior was also evident in our findings, where the APs-eugenol combination significantly outperformed single treatments and the standard chymotrypsin drug. In addition, Abdou et al. [76] evaluated two eugenol derivatives acetyleugenol and epoxyeugenol against *E. coli* and *S. aureus* and found substantial antimicrobial and anti-inflammatory activities. This supports the idea that eugenol and its derivatives can modulate microbial and immune responses, consistent with our data showing reduced cytokine levels following eugenol administration [77]. Similarly, Di Consiglio et al. [63] demonstrated that polymers with phenolic side chains, such as those found in eugenol derivatives, exhibited both antimicrobial and anti-inflammatory effects, particularly against *S.epidermidis*, further confirming the dual functionality of such compounds. Taken together, the current findings strongly support the anti-inflammatory potential of both APs and eugenol and reveal a significant enhancement in therapeutic efficacy when used in combination.

### 4.5 Antioxidant mechanisms through radical scavenging assays (DPPH Assay)

**Fig. 7:**
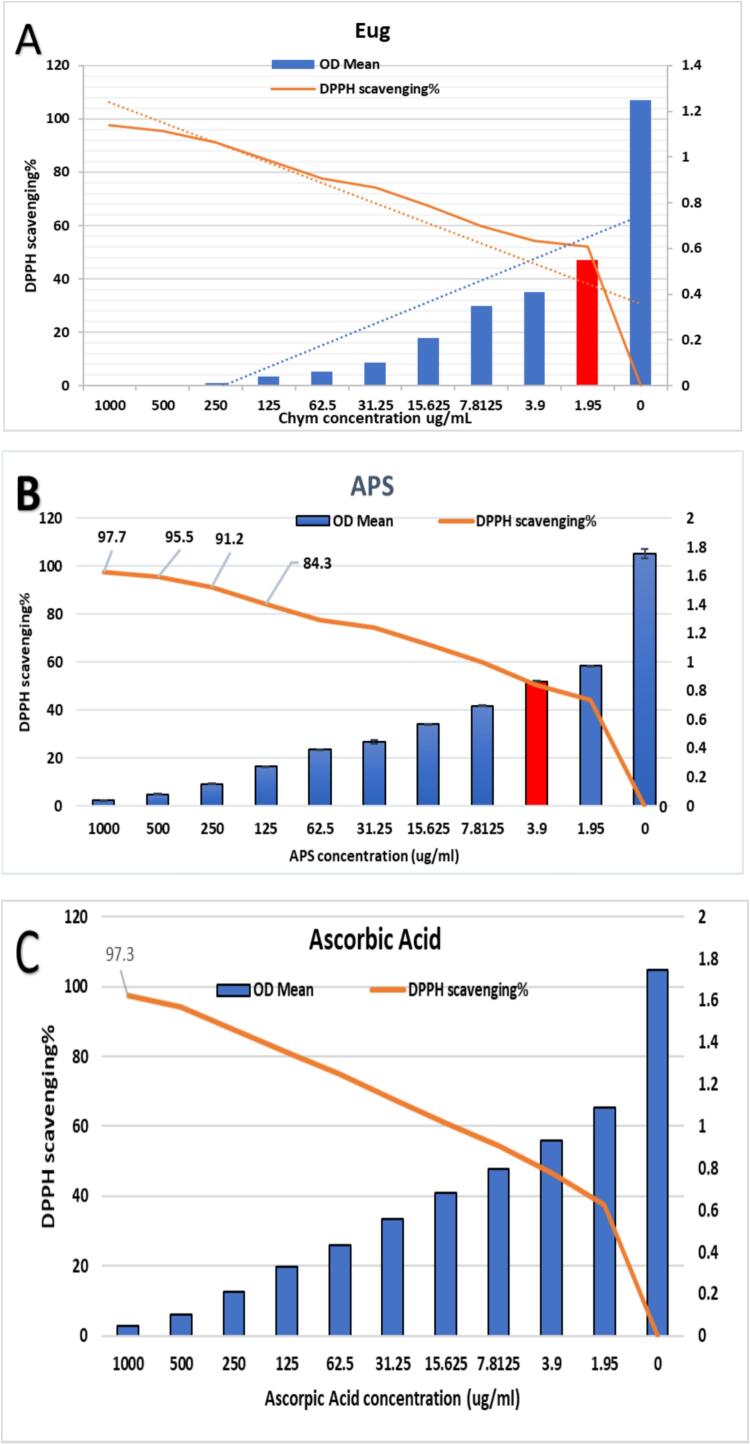
DPPH scavenging activity (%) of eugenol and APS, determining their IC_50_ values, with ascorbic acid as a reference standard. Data represent mean ± SE of three independent experiments (n = 3). Statistical significance among treatments was assessed using one-way ANOVA followed by Tukey’s post hoc test (p < 0.05).

**Fig. 8:**
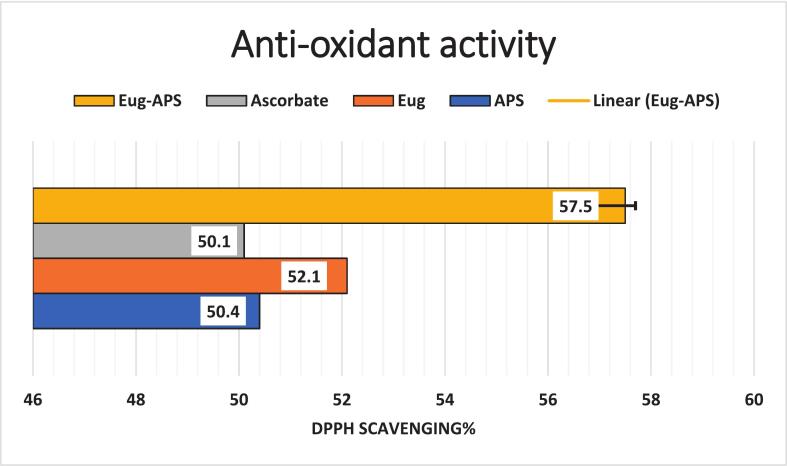
Antioxidant potential of eugenol, APS, and their combination evaluated by DPPH scavenging activity (%) at their respective IC_50_ values, compared with ascorbic acid as a reference standard. Data represent mean ± SE of three independent experiments (n = 3).

The antioxidant capacity of APS, eugenol, and their combination was quantified using the DPPH radical scavenging assay, with IC_50_ values calculated as indicators of hydrogen atom and electron donation efficiency (Fig. 7 and Fig. 8). All experiments were performed in triplicate (n = 3), and data are presented as mean ± SE. Statistical significance among treatments was assessed using one-way ANOVA followed by Tukey’s post hoc test (p < 0.05). Eugenol exhibited the highest scavenging activity with an IC_50_ of 1.95 ± 0.05 µg/mL (52.1 ± 1.2 %, a), followed by APS at 3.9 ± 0.08 µg/mL (50.4 ± 1.0 %, b). Ascorbic acid, used as a positive control, showed an IC_50_ of 5.43 ± 0.12 µg/mL (50.1 ± 0.9 %, b). Notably, the combination of APS and eugenol (1:1 v/v at their IC_50_ concentrations) demonstrated the highest antioxidant effect, reaching 57.5 ± 1.3 % DPPH scavenging activity (c), suggesting a possible synergistic interaction between APS and eugenol.

These findings indicate that eugenol possesses a higher antioxidant potential compared to APS and ascorbic acid. The enhanced activity in the combination treatment suggests a possible synergistic interaction between APs and eugenol. This observation aligns with the findings of Nurhadi et al. [78], who reported that clove bud extract, rich in eugenol and related compounds like eugenyl acetate and β-caryophyllene, exhibited very strong antioxidant activity with an IC_50_ of 9.31  ppm. The potent effect was attributed to the presence of hydroxyl groups in eugenol, which can donate hydrogen atoms to neutralize free radicals [79]. These findings indicate that eugenol exhibits superior antioxidant properties compared to APs and even the reference antioxidant, ascorbic acid [80]. This is consistent with several recent studies. For instance, For instance, Kiki, [81], reported that EOCa, a eugenol-based compound, exhibited potent antioxidant activity with an IC_50_ value of 50 µg/mL, although our results show an even stronger activity at lower concentrations, possibly due to the purity or structural variation of eugenol used. Similarly, Pires Costa et al. [82] highlighted the significant antioxidant role of eugenol derivatives through stabilization of free radicals and inhibition of oxidative chain reactions.

The moderate antioxidant activity of APs is also supported by prior reports. For example, Di Consiglio et al. [63] demonstrated that APs-based materials, such as poly (HEMA-EU), display radical scavenging properties due to the presence of phenol and acrylate groups capable of donating electrons. Although APs was less potent than eugenol, its activity (IC_50_ = 3.9 µg/mL) remained superior to ascorbic acid, suggesting that APs could contribute to antioxidant defense systems. The enhanced scavenging activity observed in the APS-eugenol combination suggests a synergistic interaction. This synergism likely results from cooperative mechanisms such as regeneration of active antioxidant forms or improved radical stabilization, which has been reported in other studies involving combinations of natural antioxidants [83]. For example, Chen et al. [84] found that combining phenolic compounds led to improved antioxidant outcomes via redox interactions and enhanced hydrogen atom transfer mechanisms. Therefore, the combined use of APs and eugenol not only amplifies antioxidant activity but also opens possibilities for designing effective antioxidant formulations using natural, biocompatible components. These results provide a scientific basis for utilizing such combinations in food preservation, pharmaceutical preparations, or biomedical applications where oxidative stress is a concern.

### 4.6 Cyclooxygenase isoforms inhibition (COX-1 and COX-2) as a molecular target of APs and eugenol

**Fig. 9:**
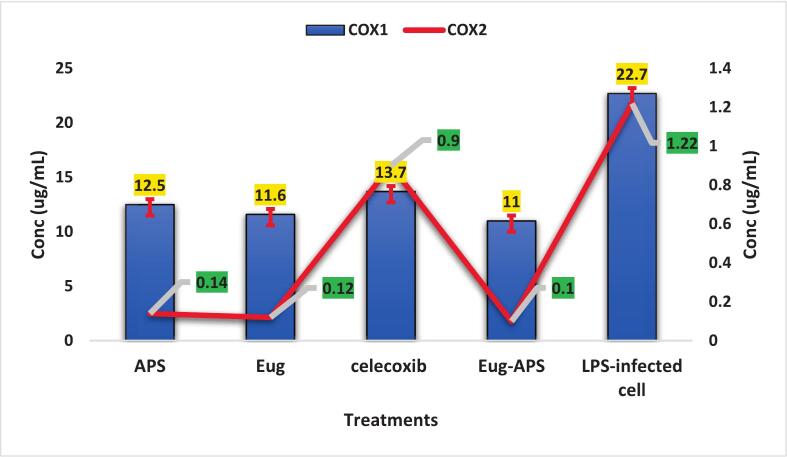
Anti-inflammatory potential of APS, eugenol, and their combination evaluated by IC_50_ values against COX-1 and COX-2. Data represent mean ± SE of three independent experiments (n = 3). Statistical significance among treatments was assessed using one-way ANOVA followed by Tukey’s post hoc test (p < 0.05).

Biochemical inhibition of cyclooxygenase isoforms was evaluated to further elucidate the molecular anti-inflammatory mechanism of APs and eugenol. The inhibitory effects of APS, eugenol, and their combination on cyclooxygenase isoforms (COX-1 and COX-2) were evaluated to elucidate their molecular anti-inflammatory potential (Fig. 9). All experiments were performed in triplicate (n = 3), and data are presented as mean ± SE. Statistical significance was assessed using one-way ANOVA followed by Tukey’s post hoc test (p < 0.05). Both APS and eugenol demonstrated notable inhibitory activity against COX enzymes, with IC_50_ values of 64.1 ± 1.2 µM (a) and 55.4 ± 1.0 µM (b), respectively, compared to the reference drug celecoxib (25.4 ± 0.8 µM, c). Notably, the combination of APS and eugenol enhanced inhibition, particularly against COX-2, achieving an IC_50_ of 0.10 ± 0.01 µg/mL (d), compared to APS (0.14 ± 0.01 µg/mL, c), eugenol (0.12 ± 0.01 µg/mL, c), and celecoxib (0.9 ± 0.05 µg/mL, b). For COX-1, the combination also yielded the lowest concentration (11 ± 0.5 µg/mL, b), outperforming APS (13.7 ± 0.6 µg/mL, a) and eugenol (12.5 ± 0.5 µg/mL, a), indicating selective COX-2 inhibition.

This finding suggests the potential for enhanced anti-inflammatory action with reduced side effects, since selective COX-2 inhibition is associated with fewer gastrointestinal risks [85]. These results are supported by earlier findings. Wang et al. [86] demonstrated that eugenol alleviates inflammation in rheumatoid arthritis by inhibiting COX-2 expression and the NF-κB signaling pathway in synovial fibroblasts which eugenol exhibited COX-2 selective inhibition with an IC_50_ of 10  µM, consistent with our findings. Similarly, Liñán-Atero et al. [87] found that clove extract reduced COX-2 expression in a carrageenan-induced paw edema model, attributing the activity to eugenol and β-caryophyllene. In another study, Damasceno et al. [77]confirmed that the hydroxyl group in eugenol plays a key role in H^+^ donation, contributing to both antioxidant and anti-inflammatory activity by neutralizing reactive species that promote COX enzyme expression. The present results also align with those of Kiki, [81], who demonstrated that essential oils rich in eugenol exert strong inhibitory effects on COX enzymes. The enhanced activity of the APs-eugenol combination could be due to the phenolic content of both agents, leading to increased radical scavenging and inflammatory mediator suppression. Overall, these findings highlight the potential therapeutic synergy between APs and eugenol, suggesting their application as a natural anti-inflammatory alternative, especially in formulations aimed at targeting COX-2–mediated inflammation without eliciting COX-1–related side effects.

### 4.7 Molecular docking

#### 4.7.1 Visualization of docking results

Molecular docking, a computer-aided drug design approach, is a valuable tool for assessing protein–ligand interactions and identifying potential therapeutic candidates. In this study, docking simulations were performed to evaluate the inhibitory potential of eugenol against five target proteins (PDB IDs: 5L3J, 5FSA, 5KIR, 3B9J, and 2XYP). As shown in Table 3, eugenol exhibited strong binding affinities, particularly with sterol demethylase (5FSA), COX-2 (5KIR), and xanthine oxidase (3B9J). The calculated binding energies ranged between –4.50 and –5.25 kcal/mol, indicating favorable and spontaneous interactions within the active sites of the selected receptors.

**Table 3:** Docking results of eugenol against target proteins (PDB IDs: 5L3J, 5FSA, 5KIR, 3B9J and 2XYP) active spots.

Eugenol						
The target protein PDB	Binding energy ΔG (Kcal/Mol)	Ligand	Bonded residues	Interaction	Distance (in A^o^ from main residue)	E (Kcal/mol)
5L3J	−4.62	6-ring	HIS 38	pi-pi	3.95	−0.7
5FSA	−5.05	O 8 6-ring	GLU 73 SER 412	H-donor pi-H	3.42 4.46	−0.6 −0.6
5KIR	−5.09	6-ring 6-ring	CYS 47 PRO 153	pi-H pi-H	3.54 4.00	−1.3 −0.6
3B9J	−5.25	O 8 6-ring	GLU 267 THR 354	H-donor pi-H	2.93 4.27	−4.3 −0.8
2XYP	−4.50	6-ring	HIS 121	pi-pi	3.71	−0.5

**Fig. 10:**
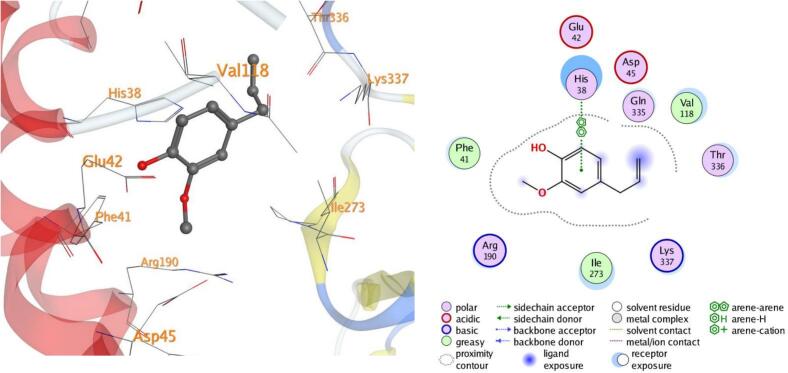
3D and 2D of docked eugenol into the active site of DNA Gyrase B (PDB ID: **5L3J**).

**Fig. 11:**
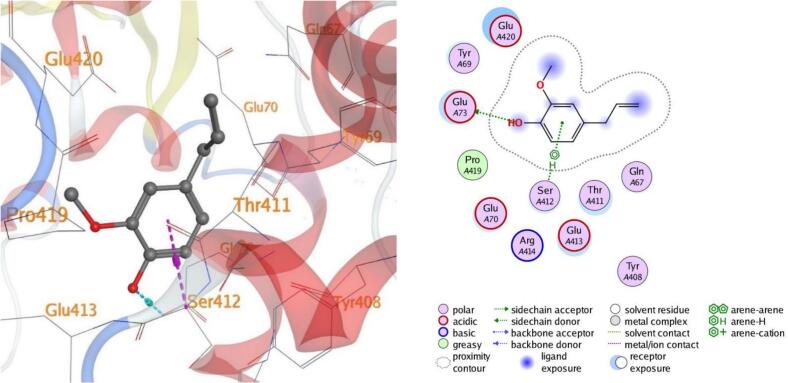
3D and 2D of docked eugenol into the active site of Sterol demethylase (PDB ID: **5FSA**).

**Fig. 12:**
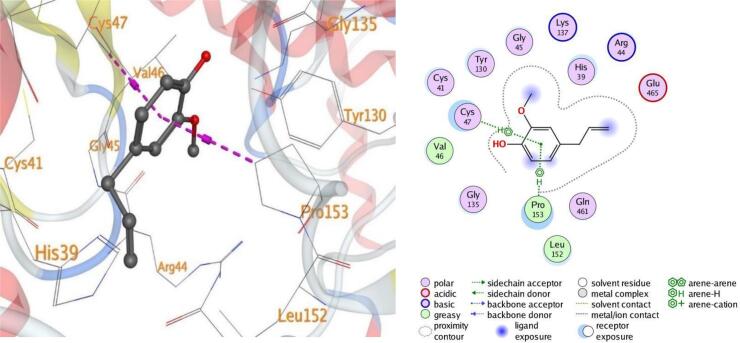
3D and 2D of docked eugenol into the active site of Cyclooxygenase-2 (COX-2)(PDB ID: **5KIR**).

**Fig. 13:**
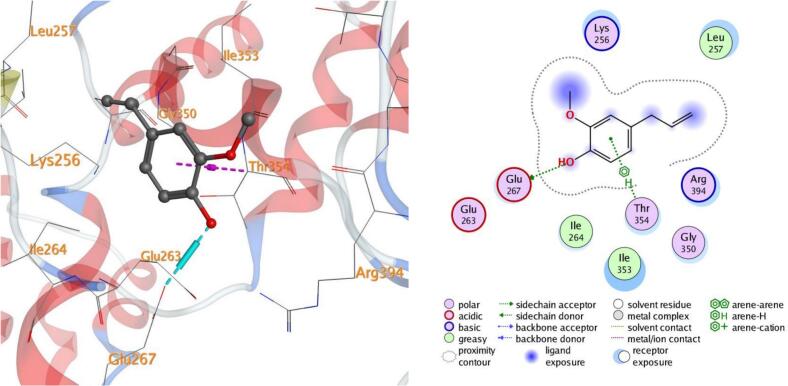
3D and 2D of docked eugenol into the active site of Xanthine oxidase (PDB ID: **3B9J**).

**Fig. 14:**
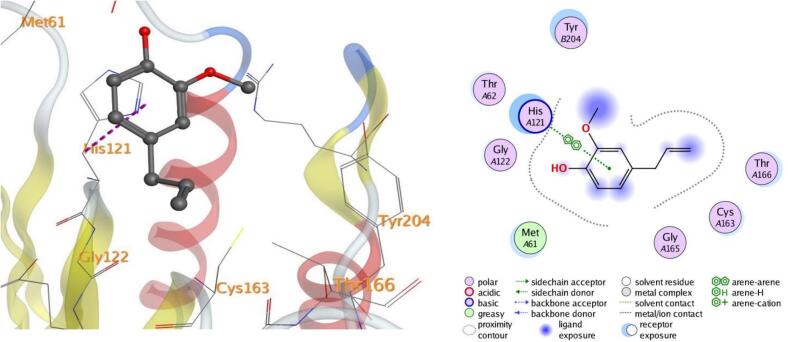
3D and 2D of docked eugenol into the active site of Caspase-3 (PDB ID: **2XYP**).

In all the docked processes the eugenol phenyl ring forms pi-pi stacked interactions and arene-H contacts with amino acid residues as HIS 38, HIS 121, SER 412, CYS 47, PRO 153, and THR 354; respectively. Also, the eugenol hydroxyl oxygen forms hydrogen bond with the residues GLU 73, and GLU 267 for the target five proteins (Fig. 10, Fig. 11, Fig. 12, Fig. 13, Fig. 14**)**.

### 4.8 Ros-mediated apoptosis and cell cycle arrest in HepG2 cells

#### 4.8.1 Dose-dependent cytotoxic and anti-proliferative effects of eugenol on HepG2 liver cancer cells

**Fig. 15:**
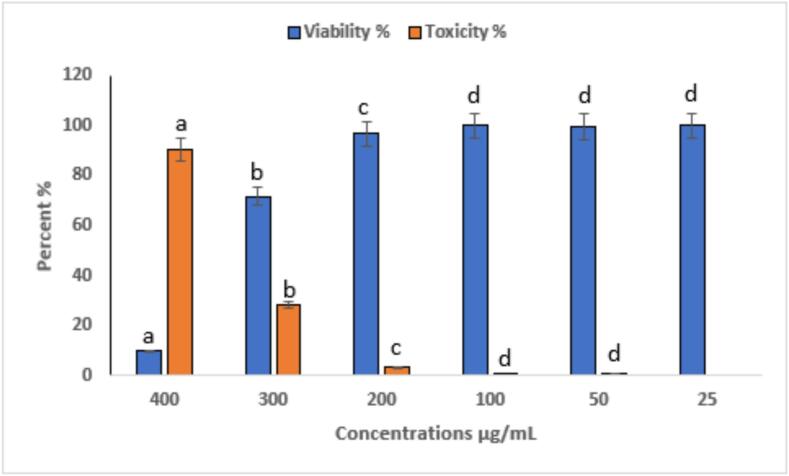
Cytotoxic activity of eugenol on HepG2 cells at different concentrations (25–400 µg/mL). Cell viability was determined using the MTT assay after 24 h of treatment. Data are presented as mean ± SE of three independent experiments (n = 3). Different letters above the bars indicate statistically significant differences among treatments (p < 0.05, one-way ANOVA followed by Tukey’s post hoc test).

**Fig. 16:**
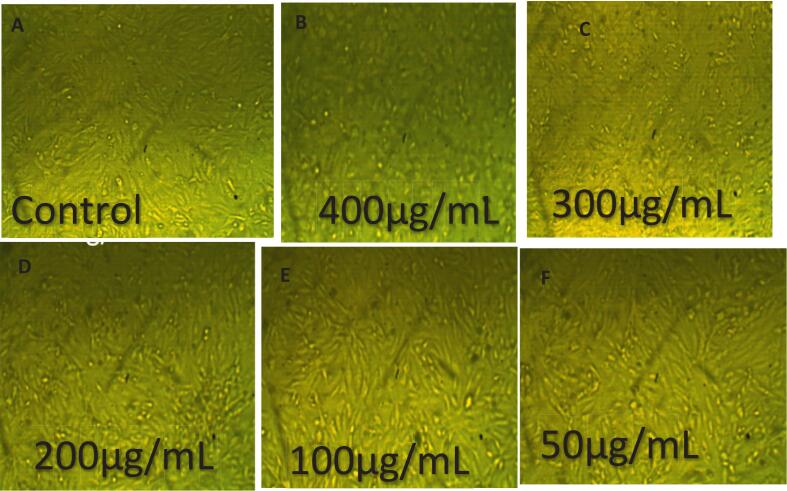
(A) Untreated control showing healthy, adherent HepG2 cells with intact morphology. (B–F) Cells treated with eugenol at concentrations of 25, 50, 100, 200, and 400 µg/mL, respectively. Images captured using an inverted microscope at 20 × magnification.

The cytotoxic and anti-proliferative effects of eugenol on HepG2 liver cancer cells were evaluated at concentrations ranging from 25 to 400 µg/mL (Fig. 15 and Fig. 16). All experiments were performed in triplicate (n = 3), and data are presented as mean ± SE. Statistical significance among concentrations was assessed using one-way ANOVA followed by Tukey’s post hoc test (p < 0.05). Eugenol exhibited a clear concentration-dependent cytotoxic effect. At the highest concentration tested (400 µg/mL), cell viability decreased to 9.77 ± 0.5 % (toxicity: 90.23 ± 0.5 %, a), whereas at lower concentrations (25–300 µg/mL), cell viability remained above 70 %, reaching 100 ± 1.2 % at 25 µg/mL (c). These findings suggest that eugenol significantly inhibits HepG2 cell proliferation at high concentrations while exhibiting minimal cytotoxicity at lower doses. Fig. 16 demonstrates the dose-dependent anticancer effect of eugenol on HepG2 hepatocellular carcinoma cells. As the concentration of eugenol increases from 50 µg/mL to 400 µg/mL, a marked reduction in cell density is observed compared to the control group, indicating a strong cytotoxic effect. These results suggest that eugenol exhibits potential anticancer activity by inhibiting the proliferation of HepG2 cells in a concentration-dependent manner. Mekky et al. [88] evaluated the cytotoxic activity of 80 % methanolic clove extract prepared under two conditions: at room temperature with sunlight exposure (RS) and at low temperature in the dark [89]. Both extracts exhibited cytotoxic effects against MCF-7 and HepG-2 cell lines, accompanied by significant DNA fragmentation, indicating apoptosis. For MCF-7 cells, the DC extract showed cytotoxicity starting at 250 µg/mL (46.7 %) and reached 100 % at 500 and 1000 µg/mL. The RS extract showed 48.25 % cytotoxicity at 500 µg/mL and also reached 100 % at 1000 µg/mL. In HepG-2 cells, the DC extract induced 50.5 % cytotoxicity at 250 µg/mL, while the RS extract showed 17.3 % cytotoxicity at 500 µg/mL. Abbasi et al. [90] reported that both eugenol nanoemulsion and Q0-eugenol nanoemulsion showed significant antiproliferative effects (p < 0.05) against breast and liver cancer cells by inducing apoptosis through ROS-dependent pathways. In PLC/PRF/5 cells, apoptosis rates were 26.7 % (eugenol NE), 84.7 % (Q0), and 16.8 % (Q0-eugenol NE). For fibroblasts: 22.6 %, 37.8 %, and 24.4 %, respectively. In KPL1 cells: 11.1 %, 57.9 %, and 19 %, respectively. Khalil et al. [91] reported that the essential oil from Anisosciadium lanatum regulated cell proliferation and viability in HepG2 liver cancer cells at sub-lethal concentrations (10–25 μg/mL), and significantly reduced cell migration and invasion. The treatment also suppressed cancer activity by downregulating the mRNA expression of pro-apoptotic markers, including BCL-2, CASPASE-3, CYP-1A1, and NFκB.

#### 4.8.2 Dose-dependent cytotoxic and antiproliferative effects of APs on HepG2 liver cancer cells

**Fig. 17:**
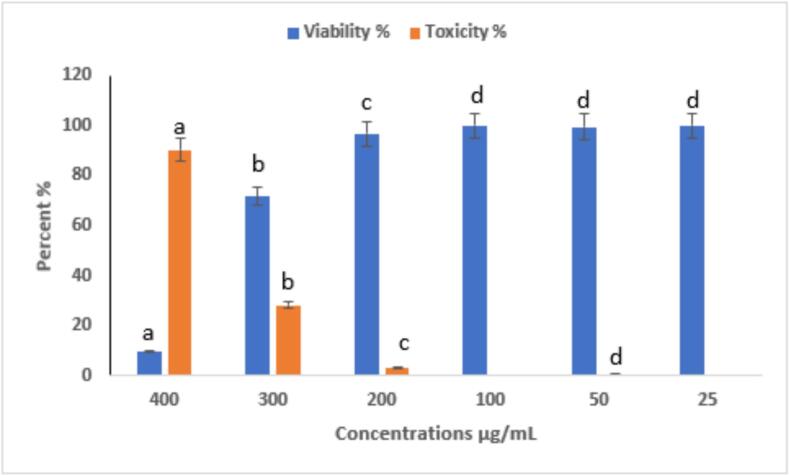
Cytotoxic activity of APs on HepG2 cells at different concentrations (25–400 µg/mL). Cell viability was determined using the MTT assay after 24 h of treatment. Data are presented as mean ± SE of three independent experiments (n = 3). Different letters above the bars indicate statistically significant differences among treatments (p < 0.05, one-way ANOVA followed by Tukey’s post hoc test).

**Fig. 18:**
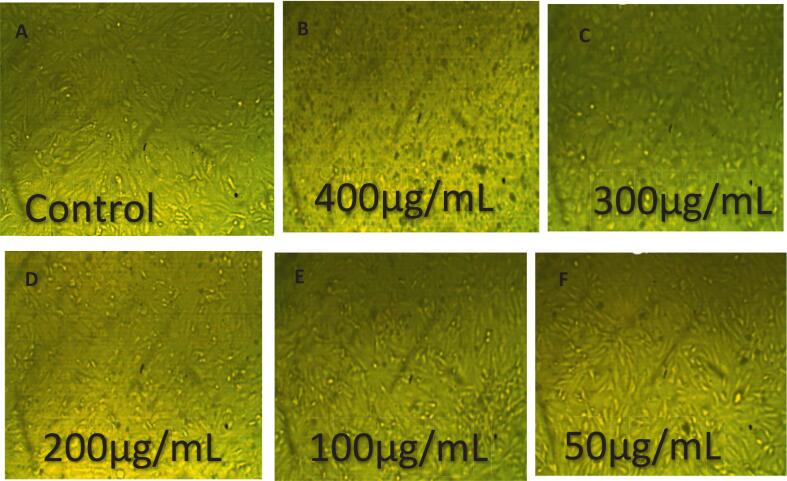
Morphological changes in HepG2 cells after treatment with APs. (A) Untreated control showing healthy, adherent HepG2 cells with intact morphology. (B–F) Cells treated with APs at concentrations of 25, 50, 100, 200, and 400 µg/mL, respectively. Images captured using an inverted microscope at 20 × magnification.

The cytotoxic and anti-proliferative effects of APs extract on HepG2 liver cancer cells were evaluated at concentrations ranging from 25 to 400 µg/mL (Fig. 17 and Fig. 18). All experiments were performed in triplicate (n = 3), and data are presented as mean ± SE. Statistical significance among concentrations was assessed using one-way ANOVA followed by Tukey’s post hoc test (p < 0.05). APs exhibited a generally weak cytotoxic effect, with significant activity only at the highest concentration tested. At 400 µg/mL, cell viability was markedly reduced to 9.77 ± 0.5 % (toxicity: 90.23 ± 0.5 %, a), whereas at 300 µg/mL, viability remained relatively high at 71.72 ± 1.2 % (b). At 200 µg/mL and lower concentrations, minimal cytotoxicity was observed, with nearly full cell survival at the lowest doses (c). These findings suggest that APs exerts an anticancer effect primarily at elevated doses. As illustrated in Fig. 18, a dose-dependent decline in cell density was observed, with significant viability loss only at higher concentrations (300–400 µg/mL), suggesting that APs exert an anticancer effect primarily at elevated doses. Previous investigations have reported comparable findings. Shen et al. [92] showed that APs inhibited premetastatic niche formation in a lung metastasis model by reducing MDSC recruitment and suppressing S1PR1/STAT3 signaling. Similarly, Jiao et al. [93] demonstrated that an Astragalus polysaccharide–nano selenium complex significantly reduced HepG2 proliferation, induced S-phase arrest, and promoted mitochondrial pathway-mediated apoptosis in vitro. Wang et al. [94] further reported that APs at 100–200 mg/L inhibited Wnt/β-catenin signaling by downregulating β-catenin, C-myc, and cyclin D1, while also reducing Bcl-2 expression and enhancing apoptosis. Notably, treatment with 400 mg/L APs for 72 h reduced HepG2 viability by over 75 %. Taken together, these data indicate that APs alone exhibits limited cytotoxicity toward HepG2 cells, with significant activity emerging only at higher concentrations. This suggests that either the active constituents are present in low abundance or their potency is relatively modest compared to conventional chemotherapeutics.

#### 4.8.3 Dose-dependent cytotoxic and antiproliferative effects of eugenol-APs on HepG2 liver cancer cells

**Fig. 19:**
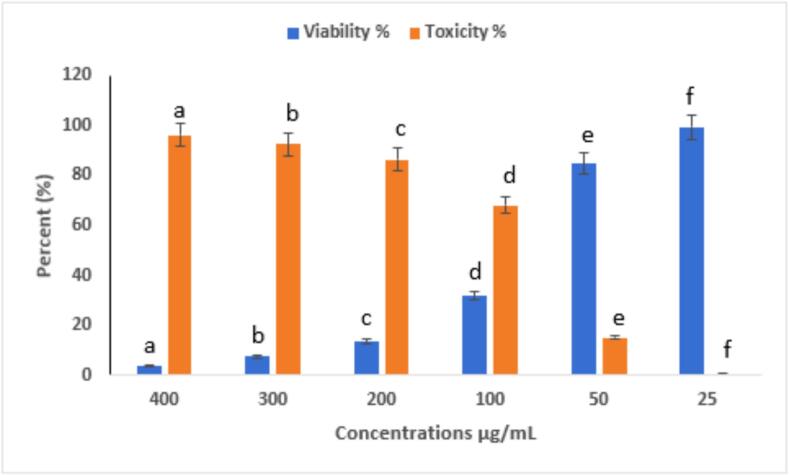
Cytotoxic activity of Eugenol-APs on HepG2 cells at different concentrations (25–400 µg/mL). Cell viability was determined using the MTT assay after 24 h of treatment. Data are presented as mean ± SE of three independent experiments (n = 3). Different letters above the bars indicate statistically significant differences among treatments (p < 0.05, one-way ANOVA followed by Tukey’s post hoc test).

**Fig. 20:**
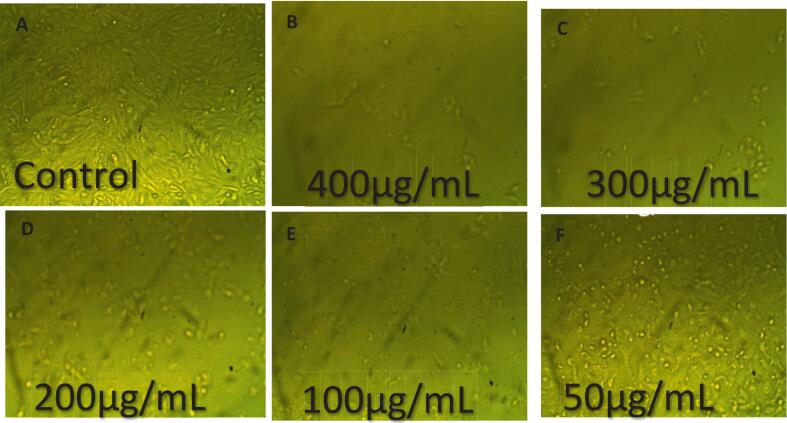
Morphological changes in HepG2 cells after treatment with Eugenol-APS. (A) Untreated control showing healthy, adherent HepG2 cells with intact morphology. (B–F) Cells treated with Eugenol-APs at concentrations of 25, 50, 100, 200, and 400 µg/mL, respectively. Images captured using an inverted microscope at 20 × magnification.

Eugenol-APs exhibited strong, dose-dependent cytotoxicity. At the highest concentration tested (400 µg/mL), cell viability decreased sharply to 3.77 ± 0.4 % (toxicity: 96.23 ± 0.4 %, a), whereas lower concentrations (25–300 µg/mL) showed progressively reduced viability, with letters above the bars indicating statistically significant differences among treatments (p < 0.05). All experiments were performed in triplicate (n = 3), and data are presented as mean ± SE. Statistical significance among concentrations was assessed using one-way ANOVA followed by Tukey’s post hoc test (p < 0.05) (Fig. 19). Morphological observations (Fig. 20) further supported these results, showing progressive cellular shrinkage, detachment, and loss of confluency. These observations strongly indicate a potent antiproliferative effect, likely attributed to the synergistic action between eugenol's phenolic structure and the delivery properties of APs. These findings suggest that Eugenol-APs exerts strong antiproliferative effects against HepG2 cells, likely due to the synergistic interaction between eugenol’s phenolic compound activity and the properties of the APs. In line with these results, previous studies have also reported the anticancer potential of eugenol. For instance, Wang et al. [95] reported that eugenol triggers apoptosis in HepG2 cells through reactive oxygen species (ROS)-mediated mitochondrial pathways. Bezerra et al. [96] emphasized its potential in cancer prevention and therapy, highlighting its dual antioxidant and pro-oxidant properties. In another study, Khaliq et al. [97] observed that eugenol displayed an IC_50_ of 14.1 µM in HL-60 cells, accompanied by activation of Caspase-3 and Caspase-9, while Hoechst 333,258 staining confirmed apoptosis through the appearance of apoptotic bodies and nuclear fragmentation. Similarly, Islam et al. [98] demonstrated that Calocedrus formosana essential oils inhibited HCT116 colon cancer cell proliferation (both p53 wild-type and null) at 20–50 µg/mL by promoting ROS generation, autophagy, and apoptosis, effects that were diminished in the presence of the ROS scavenger N-acetyl cysteine (NAC). Moreover, Zaky et al. [99] highlighted the chemopreventive role of eugenol in a DENA/AAF-induced hepatocellular carcinoma model in Wistar rats, where administration of 20 mg/kg body weight significantly improved liver histopathology and attenuated inflammation. In vitro, eugenol showed an IC_50_ of 189.29 µg/mL and inhibited HepG2 cell migration. The present study complements these findings, indicating that the APs formulation may potentiate the cytotoxic efficacy or improve the bioavailability of eugenol, thereby enhancing its therapeutic potential.

## 5 Conclusion

The collective data highlights the broad biochemical activities of APs and eugenol, both individually and in combination. Their synergistic antimicrobial activity was particularly effective against MDR clinical isolates, including *S. aureus, S. haemolyticus, E. coli, A. baumannii*, and *C. auris*, with the combination treatment showing the largest inhibition zone against S. aureus (27.3 mm). In addition to their antimicrobial properties, APs and eugenol exhibited potent antioxidant activity, as demonstrated by DPPH assays. Eugenol showed superior radical-scavenging efficiency (IC_50_ = 1.95 µg/mL) compared to APs (3.9 µg/mL) and ascorbic acid (5.43 µg/mL), while their combination achieved the highest scavenging activity (57.5 %), indicating a synergistic enhancement. Moreover, anti-inflammatory assessments revealed that the combination significantly downregulated key pro-inflammatory cytokines, including IL-6 (0.23-fold), IL-17 (0.59-fold), and TNF-α (0.36-fold), surpassing the individual effects of APs and eugenol. This was further supported by cyclooxygenase inhibition assays, where the combination exerted stronger COX-2 inhibition (0.10 µg/mL) than both individual agents and the reference drug celecoxib (0.9 µg/mL). Molecular docking studies provided mechanistic insights, demonstrating favorable binding affinities of eugenol to biologically relevant targets involved in microbial resistance and inflammation, particularly xanthine oxidase and COX-2. Finally, the combination treatment displayed a notable anticancer effect against HepG2 liver cancer cells in a dose-dependent manner, reducing cell viability by over 65 % at 400 µg/mL, indicating a strong antiproliferative potential likely attributed to synergistic mechanisms. Collectively, these findings support the development of APs–eugenol formulations as multi-targeted natural therapeutics for the integrated management of infection, inflammation, oxidative stress, and cancer.

## Ethics approval and consent to participate:

The study was conducted in accordance with the Declaration of Helsinki and approved by the Institutional Review Board (or Ethics Committee) of Alexandria University (IRB approval number and date AU13020920240133) (2 September 2024) for studies involving humans

## 8 Consent for publication

Not applicable.

## 9 Availability of data and materials

Data are available upon request from the authors.

**Clinical trial number:** Not applicable.

## CRediT authorship contribution statement

**Mohamed Khedr:** Writing – review & editing, Writing – original draft, Formal analysis, Conceptualization. **Ahmed E.M. Abdelaziz:** Validation, Conceptualization. **Fatima Albadwi:** Methodology, Data curation. **Fady Sayed Youssef:** Software, Formal analysis. **Eman M. Abd El-maksoud:** Investigation, Conceptualization. **Alsayed E. Mekky:** Software, Formal analysis. **Ebrahim Saied:** Writing – review & editing, Formal analysis, Conceptualization. **Mohamed A.M. El-Tabakh:** Writing – review & editing, Visualization. **Eslam S Abdelmouty:** Writing – review & editing, Data curation, Conceptualization. **Jayda G. Eldiasty:** Writing – review & editing, Validation, Investigation. **Mohammad Y. Alfaifih:** Software, Resources. **Ali A. Shatii:** Resources, Conceptualization. **Serag Eldin I. Elbehairii:** Formal analysis, Data curation. **Mohammed Aufy:** Writing – review & editing, Conceptualization.

## Funding

The authors extend their appreciation to the University Higher Education Fund for funding this research work under Research Support Program for Central labs at King Khalid University through the Project number (CL/CO/B/3).

## Declaration of Competing Interest

The authors declare that they have no known competing financial interests or personal relationships that could have appeared to influence the work reported in this paper.
